# Targeting Microglial Immunoproteasome: A Novel Approach in Neuroinflammatory-Related Disorders

**DOI:** 10.1021/acschemneuro.4c00099

**Published:** 2024-07-06

**Authors:** Natalia Malek, Radoslaw Gladysz, Natalia Stelmach, Marcin Drag

**Affiliations:** † Department of Chemical Biology and Bioimaging, Wroclaw University of Science and Technology, ul. Wybrzeze Wyspianskiego 27, 50-370 Wroclaw, Poland

**Keywords:** immunoproteasome, microglia, neuroinflammation, neurodegeneration, i20S inhibitors, chronic pain

## Abstract

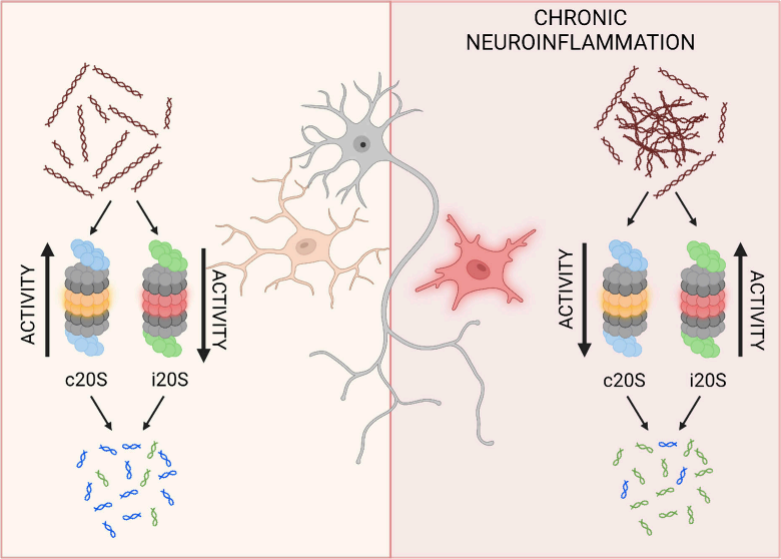

It is widely acknowledged that the aging process is linked to the accumulation of damaged and misfolded proteins. This phenomenon is accompanied by a decrease in proteasome (c20S) activity, concomitant with an increase in immunoproteasome (i20S) activity. These changes can be attributed, in part, to the chronic neuroinflammation that occurs in brain tissues. Neuroinflammation is a complex process characterized by the activation of immune cells in the central nervous system (CNS) in response to injury, infection, and other pathological stimuli. In certain cases, this immune response becomes chronic, contributing to the pathogenesis of various neurological disorders, including chronic pain, Alzheimer’s disease, Parkinson’s disease, brain traumatic injury, and others. Microglia, the resident immune cells in the brain, play a crucial role in the neuroinflammatory response. Recent research has highlighted the involvement of i20S in promoting neuroinflammation, increased activity of which may lead to the presentation of self-antigens, triggering an autoimmune response against the CNS, exacerbating inflammation, and contributing to neurodegeneration. Furthermore, since i20S plays a role in breaking down accumulated proteins during inflammation within the cell body, any disruption in its activity could lead to a prolonged state of inflammation and subsequent cell death. Given the pivotal role of i20S in neuroinflammation, targeting this proteasome subtype has emerged as a potential therapeutic approach for managing neuroinflammatory diseases. This review delves into the mechanisms of neuroinflammation and microglia activation, exploring the potential of i20S inhibitors as a promising therapeutic strategy for managing neuroinflammatory disorders.

## Introduction

A loss of proteostasis and chronic inflammation are well-documented hallmarks of aging, providing a fundamental link between neuroinflammation and neurodegenerative diseases.^1^ When the accumulation of redundant proteins surpasses their degradation, it leads to undesirable signaling and aggregation, which are key features of neuroinflammatory diseases. Consequently, cells produce nonfunctional and misfolded proteins, contributing to the onset of neurodegenerative diseases most commonly associated with aging, including Alzheimer’s disease, Parkinson’s disease, and Huntington’s disease.^2^

Proteasomes primarily function within cells to eliminate excessive proteins through the ubiquitin–proteasome system (UPS). It is generally accepted that proteasomal activity diminishes with age, and this decline has been linked to cellular senescence.^3^ Studies have shown that cells experiencing an inhibition of proteasome subunits exhibit senescence-like characteristics, underscoring the pivotal role of proteasomes in the aging process.^4^ One of the prevailing theories relating aging to oxidative stress revolves around the imbalance between the presence of reactive oxygen species (ROS) and the enzymes responsible for neutralizing them.^5,6^ As mitochondria become dysfunctional and cells are exposed to ROS, oxidized proteins start to aggregate, impairing the ability of proteasomes to process them.^6^ The 26S proteasome appears to struggle with processing oxidized proteins and, under oxidative stress, rapidly dissociates into the c20S proteasome and the 19S regulatory cap.^7^ While both the c20S and i20S proteasome subtypes are efficient at cleaving oxidized proteins, the latter seems to play a more significant role in chronic stress conditions such as neuroinflammation.^8^ Additionally, PA28 (11S), a regulatory protein associated with the immunoproteasome, enhances the activity of both constitutive and immunoproteasomes in response to oxidative stress.^9^ It is noteworthy that over time, oxidized proteins accumulate, forming large complexes, which are a characteristic feature of many neurodegenerative diseases.^10^

**Figure 1:**
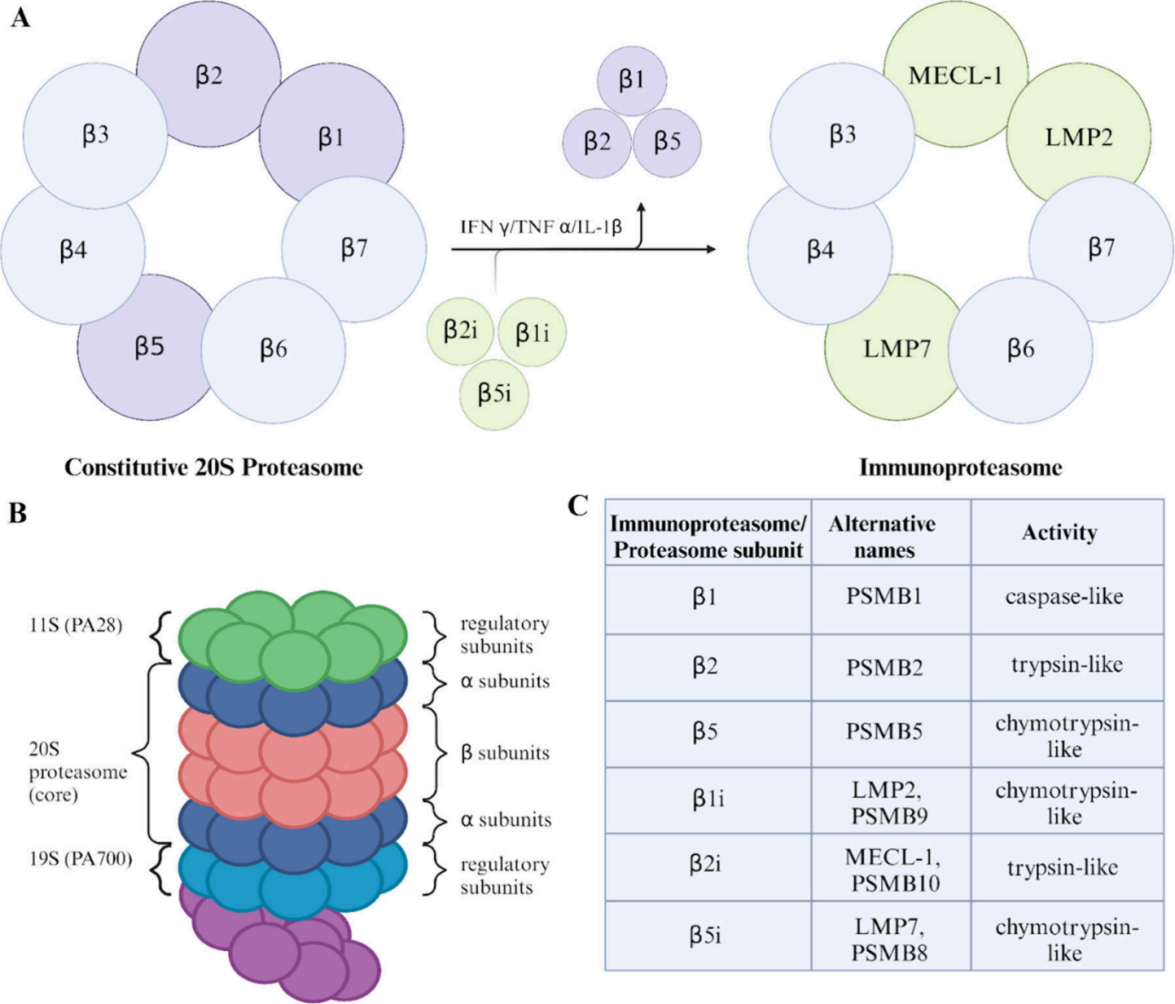
Proteasomal inner core consists of a combination of two α and two β rings, each containing either seven α or seven β subunits, respectively. While proteasomal variants share identical α rings, they differ in their β subunit composition. Upon activation by proinflammatory agents such as IFN γ, TNF α, or IL-1β, three of the β subunits within the 20S constitutive proteasome–specifically β1, β2, and β5–are replaced by their immunoproteasome counterparts, LMP2, MECL1, and LMP7, respectively (A). The proteasome consists of a 20S inner core, namely, two beta rings nestled between two alpha rings and two regulatory caps, which manifest in two distinct forms of 11S (PA28) or 19S (PA700), giving rise to possible permutations in which caps can be arranged. The immunoproteasome can have both α and β 11S caps, both 19S caps or a combination of these two types of caps (B). Three inducible beta subunits within the catalytic core possess distinct enzymatic activities, namely, the LMP2 subunit has caspase-like activity, the MECL1 subunit exhibits trypsin-like activity, and the LMP7 subunit has chymotrypsin-like catalytic function (C). Illustration created with BioRender.com.

The i20S proteolytic machine can recruit two proteasome activator regulatory components (PA28α and PA28β) and together with a catalytic core with two pairs of outer rings (seven α subunits each) and two inner rings (seven β subunits each) form a complex. The three β subunits possess different proteolytic activities: β1i (large multifunctional peptidase 2 – LMP2) – chymotrypsin-like, β2i (multicatalytic endopeptidase complex-like-1 – MECL-1) – trypsin-like and β5i (large multifunctional peptidase 7 – LMP7) – chymotrypsin-like^11^ (for more details, please see Figure 1). Interestingly, PA28α enhances the activity of immunoproteasome complex in response to oxidative stress.^9^ It is noteworthy that over time, oxidized proteins accumulate, forming large complexes, which are a characteristic feature of many neurodegenerative diseases.^10^

Neuroinflammation refers to a range of inflammatory responses that occur within the central nervous system (CNS), involving key components such as the blood–brain barrier (BBB), microglia (and other cell types that infiltrates CNS during inflammation) and neurons. While inflammatory events within the CNS naturally increase with age, they can also be triggered by tissue damage, abnormal activity of the immunological system, ischemia and more. Prolonged and excessive neuroinflammation has been linked to the development of various neurodegenerative disorders.^12−14^ i20S has been identified as a mediator of neuroinflammation through various signaling pathways, of which nuclear factor kappa B (NF-κB) is the most relevant to this condition,^15,16^ making it a promising target for potential therapeutic interventions.

Here it is worth to mention that, while the role of c20S in NF-κB signaling by degrading ubiquitinated I-κB is established,^17,18^ it remains to be defined how the i20S regulate NF-κB signaling. The reports on the i20s involvement in this pathway wellbeing remain conflicting and varies regarding the approach (genetic or pharmacological interventions), cell type and tested results.

While, studies concerned with the effect of i20S subunits on NF-κB activation yielded contradictory results,^15,19−21^ the latest data show, that i20s seems not to be involved in the regulation of canonical NF-κB, as i20S deficiency does not affect IκB-α degradation and reappearance.^22^ Nonetheless, none of the studies took under consideration redundancy of c20S and i20S systems (compensational expression of subunits and lack of selectivity of used inhibitors), thus elucidation of alternative mechanisms of NF-κB activation by i20S requires better tools and substantial research efforts in the future.

**Figure 2:**
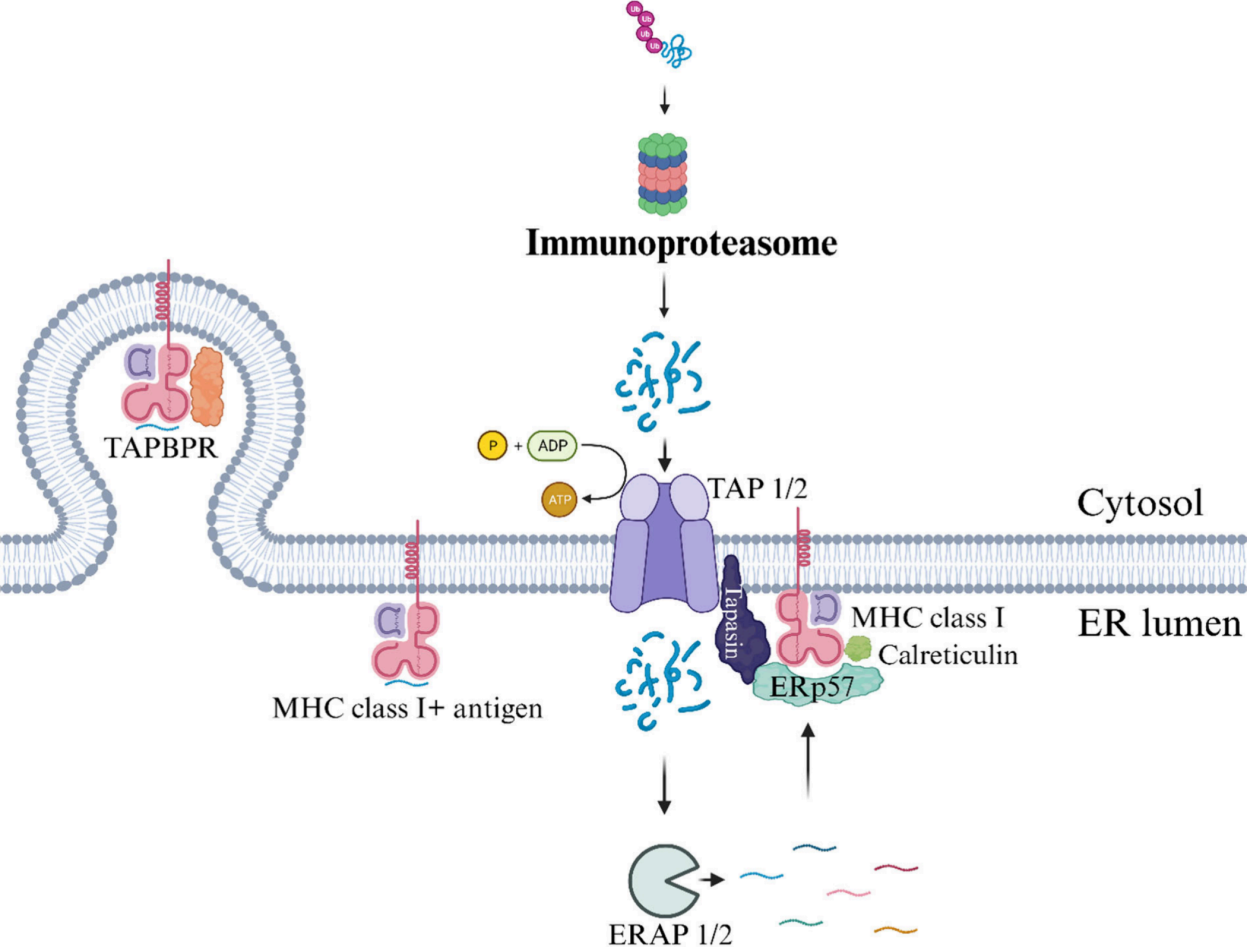
Antigen processing via the ubiquitin–proteasome system (UPS). In this illustration, the process of antigen processing through the ubiquitin–proteasome system (UPS) is depicted: (1) Immunoproteasome activity: The immunoproteasome, a specialized enzymatic complex, degrades ubiquitinated proteins, resulting in the generation of smaller peptide fragments. (2) Peptide transport to the ER: Small peptide fragments are transported to the endoplasmic reticulum (ER) lumen through the ABC transporter TAP (transporter associated with antigen processing), consisting of TAP 1 and TAP 2 proteins. (3) Antigen trimming: ER aminopeptidases (ERAPs) play a crucial role by shortening antigens to the appropriate length, making them suitable for presentation by HLA-I. (4) Loading into HLA-I: Peptides of the correct length are loaded into HLA-I-β2m (major histocompatibility complex class I) via the protein loading complex (PLC). This complex comprises TAP, the chaperone molecule calreticulin, tapasin, and ERp57. (5) Exocytosis and antigen presentation: The mature MHC I-antigen complex exits the rough endoplasmic reticulum (RER) through exocytosis, aided by the chaperone molecule TAPBPR (TAP-binding protein related). It then transits through the Golgi apparatus before reaching the cell membrane, where it exposes the antigen to CD8+ T cells (not depicted in the illustration). This illustration was created with BioRender.com.

In the nervous system, it seems that the i20S is present in various types of cells, not limited to immune cells, within the brain.^23,24^ Under normal conditions, i20S expression remains relatively low, but exposure to factors such as cytokines (e.g., interleukin-1β), interferon-γ (IFN-γ), tumor necrosis factor-α (TNF-α), or oxidative stress can significantly upregulate i20S expression.^25^ Additionally, conditions such as aging and neurodegenerative diseases also lead to heightened levels of i20S expression.^3^ This activation occurs predominantly within antigen-presenting cells (APCs) and facilitates the cleavage of proteins into shorter peptides for presentation alongside major histocompatibility complex I (MHC I) on the cell surface, which is thought to be a major function of i20S (Figure 2).^26^

As mentioned earlier, proteasomes are responsible for selectively breaking down damaged and misfolded proteins, which can occur through both ATP- and ubiquitin-dependent and -independent mechanisms. Studies have indicated that both the c20S and i20S proteasome subtypes are equally effective at degrading ubiquitinated proteins.^27^ However, they also have different functions. For instance, i20S plays a critical role in immune cells by promoting a proinflammatory environment. Additionally, i20S is more proficient than c20S in the ATP- and ubiquitin-independent degradation of oxidized proteins and proteins containing intrinsically disordered regions.^28^ Furthermore, due to the distinct catalytic activities of their subunits, which affect the variety of antigenic peptides they generate, they influence the pool of peptides presented on the cell surface by MHC class I molecules. Specifically, i20S exhibits heightened chymotrypsin activity, indicating the generation of peptides with hydrophobic or basic C-terminus, thus ideally suited for binding to MHC I.^29,30^

The enhanced turnover capacity was observed in i20S complexes compared to c20s,^8^ suggesting that the production of self-antigens in the process may also be increased. Additionally, prolonged activation of i20S could contribute to epitope spreading, a mechanism implicated in the development of autoimmune diseases. As our comprehension of the mechanism underlying the selection of peptides for presentation in MHC I class molecules remains incomplete, necessitating further investigation in the field.

Consequently, i20S dysregulation has been implicated in a multitude of diseases,^31^ including neuroinflammatory-based conditions, which are the focus of this review.

Microglia, as APCs within the CNS, serve as the first line of defense against infections and inflammation. However, during chronic inflammation, they may perpetuate a self-destructive environment by secreting inflammatory factors and presenting myelin epitopes to T cells.^32^ Additionally, microglia can present antigens to CD4+ and CD8+ T cells in response to direct inflammatory stimulation.^33^ Dendritic cells also play a role in antigen presentation within the CNS, although infiltrating dendritic cells have been found to be less efficient T-cell stimulators.^34^ Astrocytes and neurons, normally not associated with immunological responses, can still contribute to inflammation due to their abundance in the CNS. Moreover, it has been a long-standing belief that neurons do not typically display antigens, and recent findings suggest that they can be prompted to do so, especially when exposed to interferons and various stressors.^24^

It has been established that the activity of the c20S proteasome decreases with age in the nervous system.^35^ This decline has primarily been investigated in neurodegenerative disorders such as Alzheimer’s disease^35^ Parkinson’s disease.^36^ This reduction affects crucial processes in the nervous system essential for brain health, potentially contributing to age-related cognitive declines. The decrease in proteasome function with age is associated with an accumulation of oxidized proteins, leading to local inflammation.^7^ The buildup of oxidized proteins is believed to trigger a compensatory induction of the immunoproteasome (i20S).

The immunoproteasome responds to oxidative stress and is closely linked with microglial inflammatory signaling.^37^ Clinical studies also report age-related increases in immunoproteasome subunits, with significantly lower levels observed in women compared to men.^26^ Moreover, elevated levels of i20S are noted in various neurodegenerative diseases. However, whether these heightened levels contribute to impaired conditions or represent a protective response to damage remains unclear. In this discussion, we will present empirical studies indicating the potential benefits of i20S inhibitors in the mentioned disorders. Despite this, we will delve into data suggesting that the involvement of i20S could be crucial in the development of neuroinflammatory diseases and aim to persuade the reader that inhibiting this enzyme might be beneficial in unconventional treatment approaches.

## Immunoproteasome in Neuroinflammatory-Related Disorders

i20S was pointed out to take part in the CNS response to injury and neuroinflammation.^23,38^ Apart from antigen preparation, i20S is involved in limiting inflammatory damage, potentially through the removal of damaged (i.e., misfolded, oxidized) proteins and/or by regulating the profiles of cytokines produced in response to an inflammatory challenge.^8^ Studies have shown that in cells with LMP7 mutations, the basal production of interleukin-6 was significantly higher than that in control cells.^39^ Moreover, existing evidence has shown that i20S is expressed in uninjured neuronal tissues (such as the retina and brain), which also implies its role in normal function.^40^ Studies have shown that neuronal expression of MHC-I is bound by synaptic plasticity, which is crucial in the transition from acute to chronic pain states.^41,42^ Regardless of the additional roles performed by the enzyme, i20S-dependent antigen processing enables neurons to behave similar to professional APCs. Thus, following vicious cycles of inflammatory/oxidative stress in the CNS, a persistent increase in i20S may create a population of neurons susceptible to autoimmune damage. In addition, it is likely that i20S modulates neuroimmunity during T-cell trafficking to the CNS during inflammatory conditions.^43^

In summary, neuroinflammation involves a complex interplay of various cell types within the CNS, including microglia, neurons, astrocytes, and dendritic cells. The i20S play a crucial role in modulating the immune response by processing antigens for presentation. Understanding these mechanisms provides valuable insights into the pathogenesis of neuroinflammatory disorders and opens up new avenues for potential therapeutic approaches to combat these conditions. In the upcoming sections, we will explore the potential consequences of inhibiting i20S in the context of selected neuroinflammatory disorders within the CNS.

### Immunoproteasome and Alzheimer’s Disease

Alzheimer’s disease (AD) is a devastating neurodegenerative condition associated with aging, marked by the gradual deterioration of memory and cognitive abilities. The World Alzheimer Report of 2022 indicated that in that year, approximately 55 million people around the globe were affected by dementia (2019), with a significant portion having Alzheimer’s disease (from 30 to 35 million individuals). The World Health Organization projects that the worldwide dementia-afflicted population is expected to surge to 139 million by 2050^44^

AD is characterized by a relentless progression of cognitive and behavioral impairments, making it the most prevalent cause of dementia and a mounting challenge for health care systems across the world.^45^ Common symptoms of AD encompass deficiencies in short-term memory, as well as difficulties in executive functions, visuospatial skills, and apraxia.^46^ While the precise underlying mechanisms of AD remain incompletely understood, research has established a connection between the accumulation of amyloid-beta (Aβ) plaques and the formation of tau protein tangles in the brain and the development and advancement of AD.^47^ Extracellular misfolded aggregates of Aβ are thought to trigger microglial activation, leading to the release of inflammatory mediators that contribute to further disease progression.^48,49^ Studies have shown that activated microglia, in response to Aβ, transform into phagocytic cells that release proinflammatory cytokines and proteases, causing neuroinflammation and local tissue damage, which paradoxically enhances the production of Aβ.^48,50^ Systemic inflammation, often resulting from infections, may also contribute to AD progression by intensifying chronic microglial reactivity.^51^ However, the specific mechanisms and consequences of this intensification are not yet fully understood, although i20S was proposed as a potential linker in this crosstalk, as it was shown that deposits of Aβ plaques induce a long-lasting proinflammatory state in microglia that results in i20S upregulation.^52^ Researchers have found elevated activity of i20S in reactive glia around Aβ plaques in AD mice and humans. The inhibition of the LMP7 subunit of i20S through the selective inhibitor ONX-0914 resulted in a reduction in innate immune signaling molecules and receptors.^52^ Another study showed that LMP7-deficient AD mice had reduced proinflammatory cytokine levels and improved cognitive function compared to the control group.^53^ However, in both studies, inhibiting i20S or its deficiency did not affect the deposition of plaques. Moreover, increased expression of the LMP2 subunit has been observed in brain areas affected by AD in elderly patients.^13^ In recent years, mounting evidence has indicated the significant role of autoimmunity in the development and progression of AD. Additionally, viral infections, such as herpes simplex virus (HSV) and HIV-1, express immunogenic proteins similar to AD-related human proteins, potentially triggering autoimmune responses within the CNS.^54,55^ New hypotheses suggest that Aβ exhibits antimicrobial and immunomodulatory properties and is released as an early response to immune-stimulating events, but during chronic activation, Aβ contributes to autoinflammatory processes, directly connecting i20S to pathophysiology.^56,57^ Studies have already shown that suppressing i20S enhanced cognitive function in an AD mouse model of Aβ amyloidosis. This improvement occurs through the reduction of microglia-mediated inflammation, irrespective of Aβ accumulation.^58^ Other studies indicated that inhibition of both β1 and LMP2 subunits led to decrease in secretion of inflammatory cytokines from microglial cells, proposing that this intervention could present a novel therapeutic approach for AD.^59^

AD is a serious and costly health problem with tremendous effects on afflicted individuals, family members, and the global economy. Therefore, one of the largest unmet medical needs is a disease-modifying treatment for AD. Overall, the roles of microglia and i20S in AD pathogenesis present promising avenues for potential therapeutic interventions. However, further research is needed to fully understand these mechanisms and develop effective treatments for this devastating disease.

### Immunoproteasome and Parkinson’s Disease

Parkinson’s disease (PD) is a progressive neurodegenerative disorder primarily characterized by motor function impairment, but affected individuals also present with nonmotor symptoms such as autonomic dysfunction, sleep disorders, and depression. The disease is mainly associated with two major pathologies: the loss of dopaminergic neurons in specific regions of the substantia nigra and the widespread accumulation of α-synuclein (α-Syn) protein.^60^ Experimental data gathered over the years indicate that autoimmunity plays a key role in PD development.^61^ Elevated levels of innate immune factors such as the interleukins IL-1, IL-2 and IL-6 as well as TNF-α have been detected within the substantia nigra and cerebrospinal fluid of PD patients.^62,63^ Neuromelanin, the progressive loss of which is a hallmark of PD, has also been recognized as an autoantigen within the CNS. Being released from dead dopaminergic neurons, neuromelanin stimulates the maturation and functional activation of dendritic cells and then triggers an adaptive autoimmune response leading to microglial activation.^64^ In recent years, interest in the involvement of microglia in PD has increased. Current reports have shown a dual role of microglia in PD pathophysiology.^65^ The microglial NOD-like receptor family pyrin domain containing 3 (NLRP3) inflammasome that mediates caspase-1 activation and the secretion of the proinflammatory cytokines IL-1/IL-18 in response to infection, sterile inflammation or neurodegeneration may be triggered by fibrillary α-Syn.^66^ This finding was also confirmed via *in vitro* analysis of human microglia.^67^ In addition to proinflammatory signaling in PD, microglia also participate in the phagocytosis and clearance of α-Syn.^68^ PLK2, a kinase responsible for the phosphorylation of α-Syn, has also been recognized as an i20S substrate.^21^ Proteasomal activity impairment aggravates the formation of protein deposits in PD models. However, α-Syn is also responsible for the downregulation of i20S.^69^ α-Syn suppresses the expression of POMP, a chaperone responsible for i20S assembly and maturation. Induced expression of POMP enhances proteasomal activity and PLK2 degradation, alleviating α-Syn aggregation and neuronal loss in PD.^69^ Recently studies emerged directly connecting increased expression of PSMB8 with development of PD.^70^ Thus, therapies based on the intensification of i20S assembly seem to be a promising approach in novel PD treatment.

### Immunoproteasome and Chronic Pain

Chronic pain is considered to be a CNS disorder characterized by a maladaptive experience with no beneficial biological significance and involves spontaneous and evoked pain in response to noxious (hyperalgesia) or innoxious (allodynia) stimuli.^71^ Chronic pain involves structural and functional changes in pain-processing pathways and circuits located in multiple brain regions.^72^ Despite its neuronal origin, great progress has been made to demonstrate the critical roles of glial and immune cells in the pathogenesis and resolution of chronic pain, especially at the entry to the CNS from the periphery and in the CNS itself, where immune cells communicate to neurons.^73^ It was shown in a neuropathic pain model that nerve injury upregulates the immunological circuit that may be responsible for contributing to the neuroinflammation that persists for a long duration, even when the initial injury or disease subsides.^74^ Microglia are thought to have a more important role in the development of chronic pain than other immune cells, as their activation is assumed to be a key mechanism underlying the pathogenesis of chronic pain.^75^ Activated microglia can release a variety of factors, including proinflammatory cytokines (e.g., interleukins 1β and 6), brain-derived neurotrophic factors (BDNFs), and proteases, that can signal to neurons, thus enhancing neuronal firing.^75^ However, research has shown that the activation of microglia also leads to some beneficial outcomes, as released anti-inflammatory factors (e.g., interleukins 4 and 10) protect against neurotoxicity.^76^ Although microglia are able to release signaling molecules involved in the regulation of neuronal plasticity, molecules of neuronal origin also control microglial process motility in an activity-dependent manner.^77^ These bidirectional interactions affect the processing of pain, although further studies are needed to elucidate the role of neuroimmune crosstalk in the development and maintenance of chronic pain.

Clinical studies have shown a connection between autoantibodies and neuropathic pain, suggesting that these autoantibodies may be important drivers of neuropathic pain and target both neuronal and nonneuronal antigens within the CNS.^32^ Given that i20S is closely associated with the generation of autoantigens, the modulation of its catalytic subunit activity could serve as a preventive measure against the emergence of chronic pain triggered by autoimmune activation. Targeting proteasomes in the prevention and/or treatment of chronic pain is not a new concept and has already been studied in the past, giving promising results.^78,79^ However, human c20S inhibitors also induce severe toxicities and adverse effects, limiting their clinical application, including peripheral neuropathy.^80^ The development of selective inhibitors of i20S subunits created opportunities to elucidate the involvement of this enzyme in chronic pain development. Studies on MG132, a potent and nonselective inhibitor of both c20S and i20S, showed a reduction in inflammatory and neuropathic pain.^79,81^ Nonetheless, prolonged treatment with MG132 led to neuronal apoptosis, preventing its use in long-term treatment.^82^ Therefore, the ongoing advancements in the realm of i20S inhibitors suggest that a suitable compound with the desired characteristics will eventually be identified.

### Immunoproteasome and Traumatic Brain Injury

Traumatic brain injury (TBI) can be described as a disruption in brain function or evidence of brain pathology caused by an external force.^83^ Examples of external forces include a strike to the head, brain penetration by a foreign object, or rapid acceleration and deceleration of the brain. TBI is a major cause of morbidity and disability worldwide, affecting approximately 1.7 million people in the U.S. each year.^84^ TBI is associated with long-term cognitive deficits, such as memory and attention impairment, emotional instability, and sensorimotor issues. Additionally, TBI is a high risk factor for the development of other neurological conditions, including Alzheimer’s disease (AD), Parkinson’s disease (PD), epilepsy, and stroke.^85^ Although the damage caused by an external force may be irreversible, the subsequent neuroinflammatory response presents a promising target for novel TBI therapies.

Neuroinflammation triggers a rapid transformation of microglia from their resting state into a reactive proinflammatory state. This shift leads to increased expression of immunoproteasome catalytic subunits.^37^ In experimental models of controlled cortical impact (CCI) injury, treatment with an i20S inhibitor suppressed microglia-mediated inflammation by reducing IFNγ-dependent NO (nitric oxide) production.^37^ Additionally, i20S inhibition resulted in decreased phagocytosis and microglia priming. Similarly, the use of bortezomib attenuated the neuroinflammatory response, reduced functional deficits, and improved spatial learning ability in CCI models.^86^ Furthermore, bortezomib treatment significantly reduced the loss of neurons in the dentate gyrus and the hippocampal CA3 region. These findings suggest that targeting neuroinflammation through i20S modulation holds potential for the development of effective TBI therapies.

## Immunoproteasome Inhibitors in the Treatment of Neuroinflammatory Diseases

Controlling the expression and activity of the immunoproteasome represents a potent means of influencing various aspects of cell function, encompassing cellular metabolism, differentiation, and immune modulation. It is worth mentioning that inhibiting the i20S may not globally inhibit protein degradation, thus avoiding protein aggregation, including amyloids, tau proteins and others associated with neuroinflammatory diseases. This differs from inhibiting the constitutive proteasome, which leads to protein accumulation within cells. Presently, immunoproteasome inhibitors are widely available, and they are extensively used in managing numerous conditions, including hematopoietic malignancies, colitis, transplantation rejection and rheumatoid arthritis.^87,88^ Additionally, autoimmune diseases and neuroinflammatory disorders could be areas where the modulation of immunoproteasome activity holds significant therapeutic potential.

**Figure 3:**
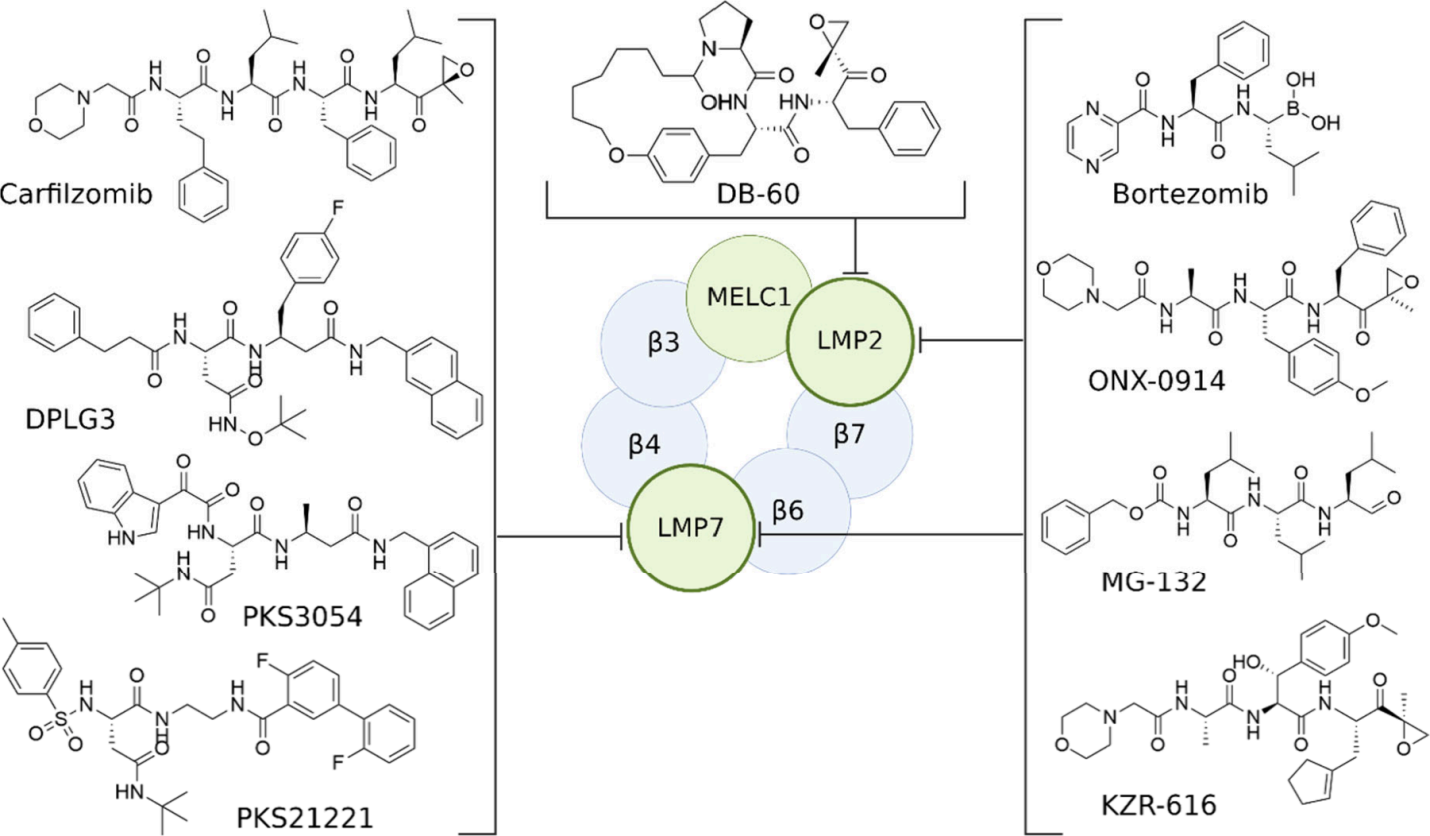
Human immunoproteasome β subunits have been the focus of considerable pharmacological interest. The initial generation drug, bortezomib, primarily employed as a c20S proteasome inhibitor, also acts as a nonspecific inhibitor of the i20S subunits LMP2 and LMP7. MG-132, a proteasomal inhibitor in the form of a peptide aldehyde, binds to the LMP2 and LMP7 subunits. Carfilzomib, based on epoxyketones, inhibits the LMP7 subunit of the immunoproteasome. The discovery of epoxyketones paved the way for the development of specific immunoproteasome inhibitors, namely ONX-0914 and zetomipzomib, both targeting the LMP5 and LMP7 subunits, as well as macrocyclic peptide epoxyketones such as DB-60. Recently noncovalent i20S subunits inhibitors came into picture (PKS3054, PKS21221, DPLG3) as promising therapy targets. The illustration was created using BioRender.com.

Human proteasome inhibitors have long been an interesting pharmacological target, and the first of them (bortezomib) was approved for the treatment of refractory myeloma at the beginning of the century.^89^ Bortezomib is a boronic acid-based dipeptidyl derivative targeting both c20S and i20s subunits. It forms a reversible covalent boron oxygen bond with the free hydroxyl of the Thr residue of the β5c subunit; however, it also exhibits inhibitory activity against the β1c, LMP2 and LMP7 subunits.^90^ The use of bortezomib has expanded to various immunological-based disorders, as it exhibits strong anti-inflammatory properties due to reactivity toward i20S.^91^ Despite the high efficacy of treatment, the duration of therapy had to be restricted due to serious c20S-dependent side effects, namely, the development of peripheral neuropathy.^92^ Although bortezomib-induced peripheral neuropathy (BIPN) is one of the most troubling adverse reactions, the pathological mechanisms of it are yet to be fully understood.^93^ Cumulative preclinical findings indicate axonal degeneration of the sciatic nerve,^94^ impairment of mitochondrial functions^95^ and disruption in intracellular signaling^96^ among them. The success of bortezomib created the basis for the discovery and development of novel proteasome inhibitors as immunomodulatory agents (Figure 3).

The discovery of an electrophilic epoxyketone warhead allowed us to overcome limitations related to the application of boronic derivatives. Epoxyketone irreversibly bonds with the Thr1 hydroxyl group while not affecting serine or cysteine proteases. This improvement in selectivity resulted in the development and approval of the second-generation c20S inhibitor carfilzomib.^97^ Similarly to bortezomib, carfilzomib inhibits chymotrypsin-like activity of both human proteasomes. However, at therapeutic concentrations, it does not effectively inhibit trypsin or caspase-like activity while reducing chymotrypsin-like activity by more than 80% by inhibiting the β5c subunit and β5i subunit.^98^ It also induces peripheral neuropathy at lower frequency compared to bortezomib while maintaining equivalent proteasomal inhibition level.^99^ Compounds bearing epoxyketone moieties have also become a basis for the development of selective i20S inhibitors. During the development of selective inhibitors, it was suggested that each of the i20S subunits plays a distinctive role in maintaining cell homeostasis, and as LMP2 and LMP7 subunits facilitate the generation of peptides for antigen presentation, LMP7 is also responsible for the decrease in proinflammatory cytokine levels.^100^ The involvement of MECL-1 in these processes remains to be established.

The cβ1 and LMP2 subunits are the most distinguishable counterparts due to their different substrate cleavage preferences at the P1 position. While the former recognizes acidic residues (caspase-like activity), the latter has an enhanced preference for hydrophobic residues (chymotrypsin-like activity). This key difference led to the development of various LMP2-specific inhibitors, and most known compounds share some similar features in their structure. These compounds contain either Leu (UK-101, DB-60, DB-310 and ML604440), Cha (LU-001i) or Phe (KZR-504) moieties at the P1 position and a cyclic residue at the P3 position.^101,102^ Moreover, significant efforts are underway to enhance the BBB permeability and effectiveness of i20S inhibitors, suggesting that these compounds could potentially serve as novel treatments for AD.^103^ All of these compounds also contain an epoxyketone warhead, except ML604440, which is a boronic acid derivative.

Subunits cβ2 and MECL-1 share similar structures and activities, which hinders the development of selective substrates and inhibitors. The only well-described MECL-1 inhibitor thus far is LU-002i, which is a derivative of ONX-0914, another i20S inhibitor targeting LMP2/LMP7 subunits.^104^ The replacement of Phe at the P1 position with a bulkier moiety allows the enhancement of selectivity toward MECL-1.

Inhibitors of LMP7 are the most prevalent group of compounds due to the importance of chymotrypsin-like activity. Similar to cβ2/MECL-1, cβ5 and LMP7 share vast similarity in structure. The main difference is at the S1 binding pocket, which is larger in the LMP7 subunit, allowing it to accommodate bulkier hydrophobic residues such as Phe or Tyr. N,C-capped dipeptides and dipeptidomimetics have been reported to noncovalently inhibit the LMP7 subunit.^105,106^ Thiazoles containing bulky quinolone moieties that can fit into the S1 cleft have also been developed and show inhibitory properties.^107^ Covalent inhibitors have been proven to lower LMP7 selectivity and coinhibit subunits other than LMP7 due to the same catalytic mechanism of all c20S active sites. Targeting the noncatalytic residues allowed the development of LMP7-specific compounds. By forming covalent bonds with residues other than Tyr1, it is possible to subdue the coinhibition of other subunits. Cys48 was determined to be a viable nucleophilic target since c20S encodes a nonreactive Gly residue at this position. Compound PRN1126 was designed to act as a noncovalent inhibitor and proved to be selective toward human LMP7; however, it exhibited no therapeutic potency.^108^ Research focused on tripeptide analogs of carfilzomib led to the development of ONX-0914.^109^ Newly discovered compound exhibited about 15 to 40-fold selectivity for LMP7 over β5c subunit, depending on the assay and experimental system employed. Moreover, crystallographic data revealed that ONX-0914 selectivity is mainly generated by the interactions of P1 tyrosine with the S1 pocket of LMP7.^110^ Selective inhibition of LMP7 (with the use of ONX-0914) mitigated neuroinflammation with reduced microglial activation and lower inflammatory marker (TNF-α, iNOS, and COX2 and proinflammatory cytokine) production in activated microglia.^37,111^ Moreover, ONX-0914 was shown to be effective in various models of immune-dependent disorders.^112^ Still highly useful as a pharmacological tool, ONX-0914 demonstrated poor pharmaceutical properties (in particular low solubility), which prevented its transition to clinical trials. Moreover, studies have proven that the inhibition of cytokine expression requires in fact the inhibition of at least two of the three i20S subunits simultaneously: LMP7/MECL-1 or LMP7/LMP2.^113^ The coinhibition of LMP2 and LMP7 is a novel potential therapeutic strategy for the treatment of various pathologies, as it can inhibit macrophage phagocytosis and inhibit T-cell activation and differentiation *in vivo*.^108^ Efforts focused on developing LMP7/LMP2 inhibitors resulted in KZR-616 (zetomipzomib) synthesis, which is characterized by high efficacy in preclinical research and a good pharmacological profile.^113^ Studies on KZR-616 in immune diseases progressed rapidly, and KZR-616 has went through phase I clinical trials and is now being evaluated in multiple studies at the phase II level.^114^

On top of that, noncovalent i20S inhibitors have emerged as promising agents for various therapeutic applications. Among them, DPLG3, a LMP7-targeting compound, has demonstrated significant potential. It suppresses cytokine release in vitro and has shown efficacy in promoting long-term acceptance of cardiac allografts in murine models.^115^ Additionally, Singh et al. have reported a series of β5i-selective dipeptidomimetic inhibitors. One such compound, PKS3054, has been found to inhibit the activation of human CD4+ T-lymphocytes and the proliferation of T cells within peripheral blood mononuclear cells (PBMCs) at noncytotoxic concentrations.^106^ Another promising compound, PKS21221, has exhibited potent cytotoxicity against multiple myeloma cell lines such as MM.1S and RPMI 8226, as well as differentiated antibody-secreting cells (ASCs). Furthermore, PKS21221 has been shown to inhibit T cell proliferation in a dose-dependent manner, highlighting its potential as a therapeutic agent for various immune-related disorders.^116^

## Conclusions and Future Directions

In recent decades, significant strides have been taken in comprehending the role of the constitutive proteasome in neurodegenerative diseases and exploring its therapeutic potential.^117^ Nevertheless, the i20S is emerging as a promising target in disorders rooted in neuroinflammation. Moreover decreased activation of i20S caused by inhibitors may lead to increased expression of c20s subunits, that may be beneficial, as lower activity of c20s is one of major signs of aging and concomitant age-related neurological disorders. Consequently, there is a need for increased scientific endeavors to unravel the molecular basis linking immunoproteasome dysregulation to neurodegenerative diseases and shed light on its therapeutic potential. Despite the recent exploration of the impact of pharmacological immunoproteasome inhibition on neurodegeneration and its promising efficacy, clinical applications of i20S inhibitors are likely to face limitations, such as poor penetration across the BBB and a short half-life. Nonetheless, significant improvements are being made to enhance the BBB permeability of these compounds, promising future therapies.^103^

Preclinical and clinical evidence regarding the involvement of i20S in neuroinflammatory diseases is continually increasing, piquing the growing interest of the scientific community. To date, a variety of anti-inflammatory and auto modulatory inhibitors specific to i20S have been documented (Table 1). Nevertheless, most of these analogs are designed to exclusively target one of the three catalytic subunits of i20S, a limitation that impedes the effective inhibition of cytokine production. However, creating a single inhibitor capable of effectively targeting two or all three distinct immunoproteasome β-subunits remains a formidable challenge. Following the successful development of KZR-616, a dual LMP2/LMP7 selective inhibitor, it is anticipated that more selective inhibitors with clinical efficacy will emerge in the near future.

**Table 1:** Compilation of the Most Promising Immunoproteasome Inhibitors, Their Specific Target Subunits, and the Potential Therapeutic Benefits They Have Exhibited in Managing Immunological Disorders, Thus Opening Doors for Future Research and Exploration

Name	Subunits	Disorder	Citations
**bortezomib**	cβ1, LMP2, LMP7	Traumatic brain injury, lupus nephritis, myocarditis, refractory myeloma	(86, 91)
**Carfilzomib**	cβ5, LMP7	Multiple myeloma	(120)
**DB-310**	LMP2	Alzheimer’s disease	(121)
**KZR-504**	LMP2	Inflammation	(113, 122)
**KZR-616 (Zetomipzomib)**	LMP7	Inflammation, systemic lupus erythematosus	(113, 114)
**Lu-001i**	LMP2	Autoimmune diseases	(101)
**MG132**	cβ5, LMP2, LMP7	Inflammatory and neuropathic pain	(79, 81)
**ML604440**	LMP2	Autoimmune diseases	(114)
**ONX-0914**	LMP2, LMP7	Alzheimer’s disease, traumatic brain injury, ischemic brain damage, autoimmune diseases	(37, 53, 108, 112)
**DB-60**	LMP2	Alzheimer’s disease	(102)
**DPLG3**	LMP7	Cardiac transplantation	(115)
**PKS3054**	LMP7	Autoimmune diseases	(106)
**PKS21221**	LMP7	Autoimmune diseases	(116)

The conundrum lies in the fact that while proteasomes inhibition may reduce immune responses in microglia cells, it also disrupts the degradation of damaged and unfolded proteins, leading to aggregation - an underlying cause of neurodegenerative diseases. The beneficial effects of an immunoproteasome inhibitor may depend on the timing of the interference. Impaired proteasome function has been reported in the brain tissue of patients with neurodegenerative diseases, and there are indications that proteasome activators may be beneficial in such situations. Although the involvement of the 20S proteasome in neuroinflammatory diseases, along with its activators and inhibitors, has been discussed in recent reviews,^118,119^ our focus here is on the i20S. In conclusion, the emerging picture suggests that i20S is becoming an interesting target, but there is still much to be understood about its precise role in pathogenesis as well as its value as a therapeutic target. All in all, i20S subunit-selective inhibitors have the potential to revolutionize the treatment of neuroinflammatory diseases. Considering the aforementioned factors, the primary hurdle in proteasome studies lies in the development of specific tools capable of accurately distinguishing between these two enzymes. The inherent similarity between them poses a challenge in creating precise tools, and the currently available options come with limitations. At the stake there are i20S subunit-selective inhibitors have the potential to revolutionize the treatment of neuroinflammatory diseases.

To sum up. while there is a need for ongoing efforts to develop specific inhibitors tailored to target i20S and facilitate their clinical integration, this promising therapeutic approach is expected to attract growing attention.
